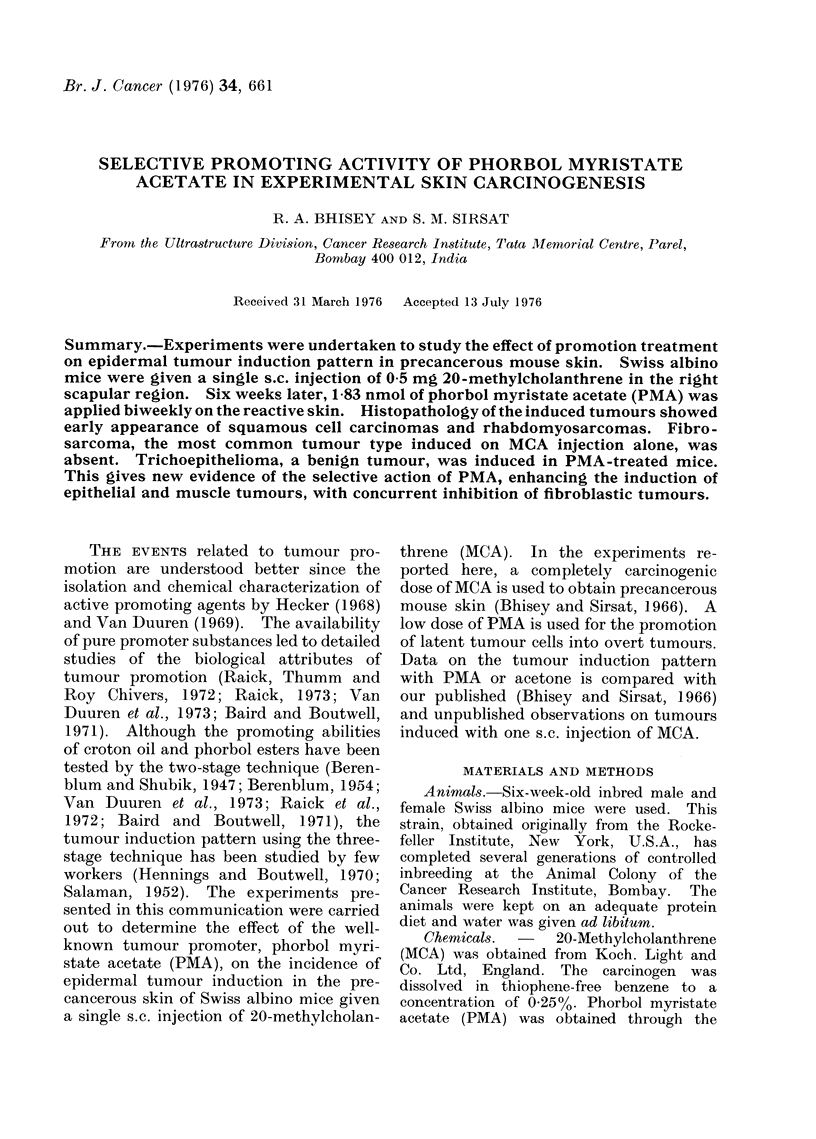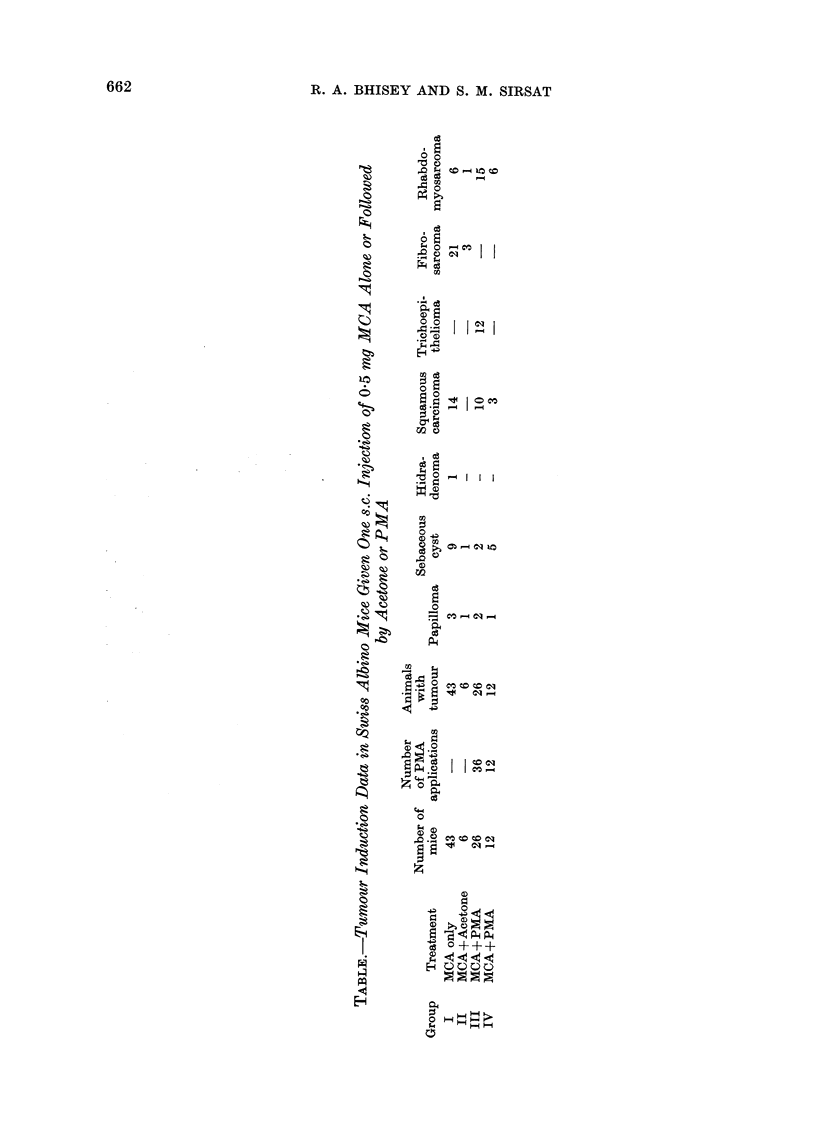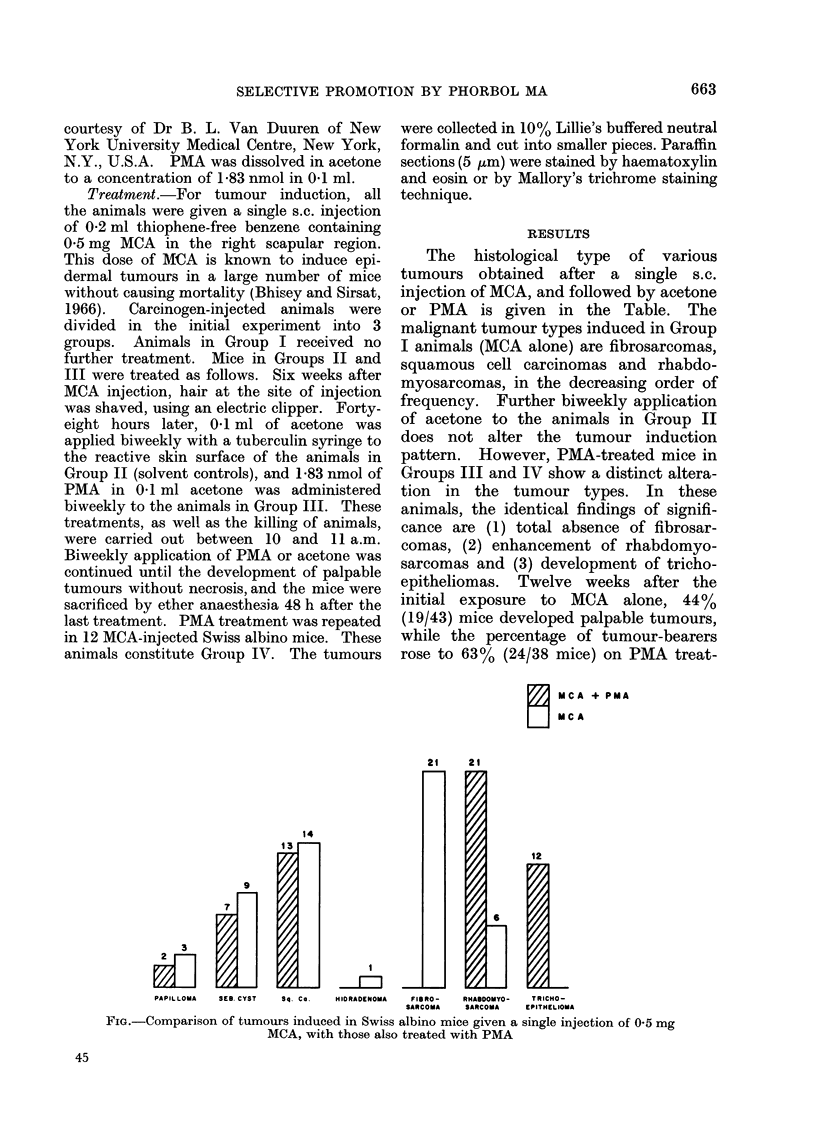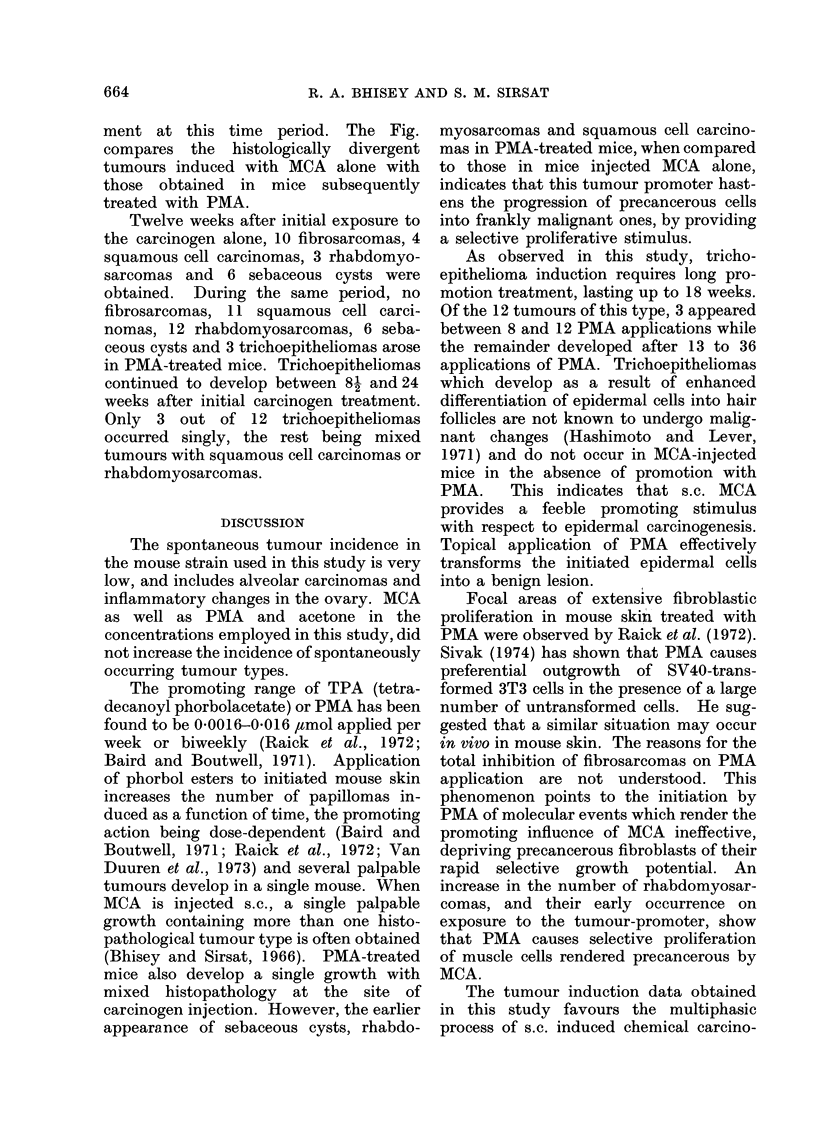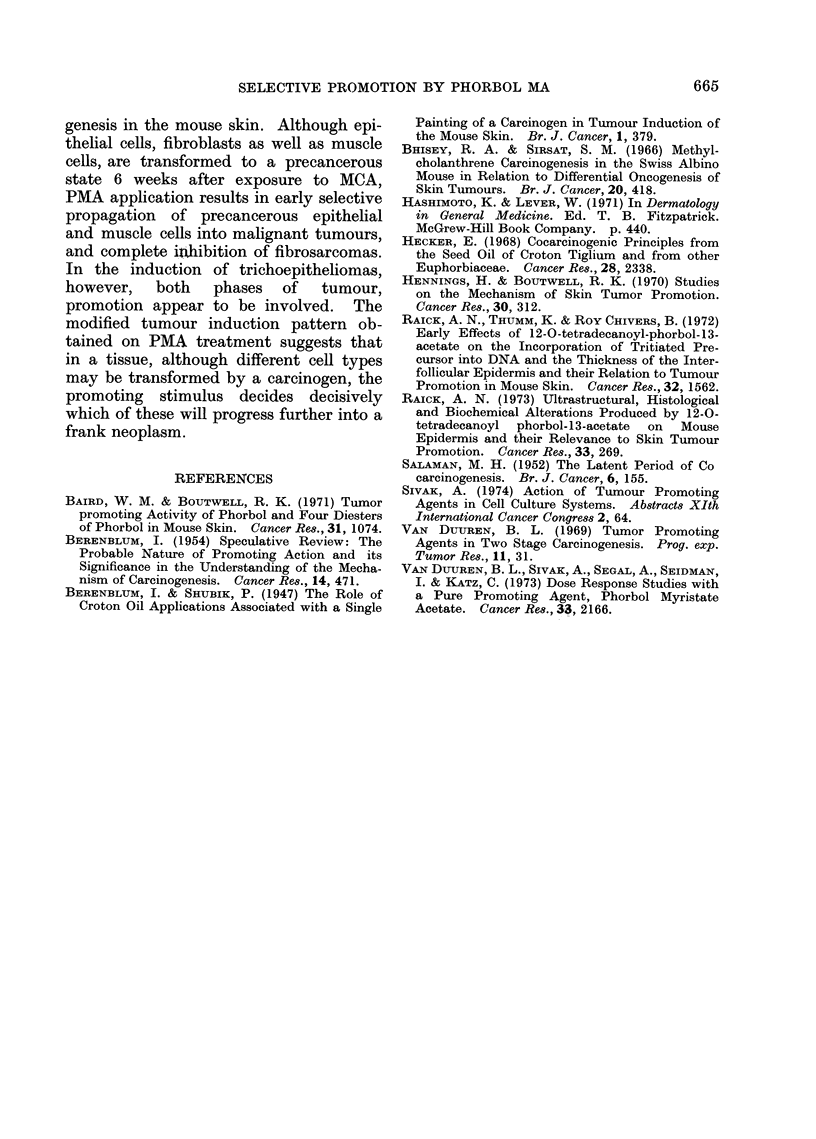# Selective promoting activity of phorbol myristate acetate in experimental skin carcinogenesis.

**DOI:** 10.1038/bjc.1976.229

**Published:** 1976-12

**Authors:** R. A. Bhisey, S. M. Sirsat

## Abstract

Experiments were undertaken to study the effect of promotion treatment on epidermal tumour induction pattern in precancerous mouse skin. Swiss albino mice were given a single s.c. injection of 0-5 mg 20-methylcholanthrene in the right scapular region. Six weeks later, 1-83 nmol of phorbol myristate acetate (PMA) was applied biweekly on the reactive skin. Histopathology of the induced tumours showed early appearance of squamous cell carcinomas and rhabdomyosarcomas. Fibrosarcoma, the most common tumour type induced on MCA injection alone, was absent. Trichoepithelioma, a benign tumour, was induced in PMA-treated mice. This gives new evidence of the selective action of PMA, enhancing the induction of epithelial and muscle tumours, with concurrent inhibition of fibroblastic tumours.


*Br. J. Cancer* (1976) **34**, 661

### SELECTIVE PROMOTING ACTIVITY OF PHORBOL MYRISTATE ACETATE IN EXPERIMENTAL SKIN CARCINOGENESIS

R. A. BHISEY AND S. M. SIRSAT

*From the Ultrastructure Division, Cancer Research Institute, Tata Memorial Centre, Parel, Bombay* 400 012, *India*

Received 31 March 1976 Accepted 13 July 1976

**Summary.—Experiments were undertaken to study the effect of promotion treatment on epidermal tumour induction pattern in precancerous mouse skin. Swiss albino mice were given a single s.c. injection of 0·5 mg 20-methylcholanthrene in the right scapular region. Six weeks later, 1·83 nmol of phorbol myristate acetate (PMA) was applied biweekly on the reactive skin. Histopathology of the induced tumours showed early appearance of squamous cell carcinomas and rhabdomyosarcomas. Fibrosarcoma, the most common tumour type induced on MCA injection alone, was absent. Trichoepithelioma, a benign tumour, was induced in PMA-treated mice. This gives new evidence of the selective action of PMA, enhancing the induction of epithelial and muscle tumours, with concurrent inhibition of fibroblastic tumours.**

THE EVENTS related to tumour promotion are understood better since the isolation and chemical characterization of active promoting agents by Hecker (1968) and Van Duuren (1969). The availability of pure promoter substances led to detailed studies of the biological attributes of tumour promotion (Raick, Thumm and Roy Chivers, 1972; Raick, 1973; Van Duuren *et al.*, 1973; Baird and Boutwell, 1971). Although the promoting abilities of croton oil and phorbol esters have been tested by the two-stage technique (Berenblum and Shubik, 1947; Berenblum, 1954; Van Duuren *et al.*, 1973; Raick *et al.*, 1972; Baird and Boutwell, 1971), the tumour induction pattern using the three-stage technique has been studied by few workers (Hennings and Boutwell, 1970; Salaman, 1952). The experiments presented in this communication were carried out to determine the effect of the well-known tumour promoter, phorbol myristate acetate (PMA), on the incidence of epidermal tumour induction in the precancerous skin of Swiss albino mice given a single s.c. injection of 20-methylcholanthrene (MCA). In the experiments reported here, a completely carcinogenic dose of MCA is used to obtain precancerous mouse skin (Bhisey and Sirsat, 1966). A low dose of PMA is used for the promotion of latent tumour cells into overt tumours. Data on the tumour induction pattern with PMA or acetone is compared with our published (Bhisey and Sirsat, 1966) and unpublished observations on tumours induced with one s.c. injection of MCA.

### MATERIALS AND METHODS

*Animals.*—Six-week-old inbred male and female Swiss albino mice were used. This strain, obtained originally from the Rockefeller Institute, New York, U.S.A., has completed several generations of controlled inbreeding at the Animal Colony of the Cancer Research Institute, Bombay. The animals were kept on an adequate protein diet and water was given *ad libitum.*

*Chemicals.* — 20-Methylcholanthrene (MCA) was obtained from Koch. Light and Co. Ltd, England. The carcinogen was dissolved in thiophene-free benzene to a concentration of 0·25%. Phorbol myristate acetate (PMA) was obtained through the

TABLE.—*Tumour Induction Data in Swiss Albino Mice Given One s.c. Injection of* 0·5 *mg MCA Alone or Followed by Acetone or PMA*

| Group | Treatment | Number of mice | Number of PMA applications | Animals with tumour | Papilloma | Sebaceous cyst | Hidra-denoma | Squamous carcinoma | Trichoepi-thelioma | Fibro-sarcoma | Rhabdo-myosarcoma |
|---|---|---|---|---|---|---|---|---|---|---|---|
| I | MCA only | 43 | — | 43 | 3 | 9 | 1 | 14 | — | 21 | 6 |
| II | MCA+Acetone | 6 | — | 6 | 1 | 1 | - | — | — | 3 | 1 |
| III | MCA+PMA | 26 | 36 | 26 | 2 | 2 | - | 10 | 12 | — | 15 |
| IV | MCA+PMA | 12 | 12 | 12 | 1 | 5 | - | 3 | — | — | 6 |

courtesy of Dr B. L. Van Duuren of New York University Medical Centre, New York, N.Y., U.S.A. PMA was dissolved in acetone to a concentration of 1·83 nmol in 0·1 ml.

*Treatment.*—For tumour induction, all the animals were given a single s.c. injection of 0·2 ml thiophene-free benzene containing 0·5 mg MCA in the right scapular region. This dose of MCA is known to induce epidermal tumours in a large number of mice without causing mortality (Bhisey and Sirsat, 1966). Carcinogen-injected animals were divided in the initial experiment into 3 groups. Animals in Group I received no further treatment. Mice in Groups II and III were treated as follows. Six weeks after MCA injection, hair at the site of injection was shaved, using an electric clipper. Forty-eight hours later, 0·1 ml of acetone was applied biweekly with a tuberculin syringe to the reactive skin surface of the animals in Group II (solvent controls), and 1·83 nmol of PMA in 0·1 ml acetone was administered biweekly to the animals in Group III. These treatments, as well as the killing of animals, were carried out between 10 and 11 a.m. Biweekly application of PMA or acetone was continued until the development of palpable tumours without necrosis, and the mice were sacrificed by ether anaesthesia 48 h after the last treatment. PMA treatment was repeated in 12 MCA-injected Swiss albino mice. These animals constitute Group IV. The tumours were collected in 10% Lillie's buffered neutral formalin and cut into smaller pieces. Paraffin sections (5 μm) were stained by haematoxylin and eosin or by Mallory's trichrome staining technique.

### RESULTS

The histological type of various tumours obtained after a single s.c. injection of MCA, and followed by acetone or PMA is given in the Table. The malignant tumour types induced in Group I animals (MCA alone) are fibrosarcomas, squamous cell carcinomas and rhabdomyosarcomas, in the decreasing order of frequency. Further biweekly application of acetone to the animals in Group II does not alter the tumour induction pattern. However, PMA-treated mice in Groups III and IV show a distinct alteration in the tumour types. In these animals, the identical findings of significance are (1) total absence of fibrosarcomas, (2) enhancement of rhabdomyosarcomas and (3) development of trichoepitheliomas. Twelve weeks after the initial exposure to MCA alone, 44% (19/43) mice developed palpable tumours, while the percentage of tumour-bearers rose to 63% (24/38 mice) on PMA treat-

FIG.—Comparison of tumours induced in Swiss albino mice given a single injection of 0·5 mg MCA, with those also treated with PMA

ment at this time period. The Fig. compares the histologically divergent tumours induced with MCA alone with those obtained in mice subsequently treated with PMA.

Twelve weeks after initial exposure to the carcinogen alone, 10 fibrosarcomas, 4 squamous cell carcinomas, 3 rhabdomyosarcomas and 6 sebaceous cysts were obtained. During the same period, no fibrosarcomas, 11 squamous cell carcinomas, 12 rhabdomyosarcomas, 6 sebaceous cysts and 3 trichoepitheliomas arose in PMA-treated mice. Trichoepitheliomas continued to develop between 8½ and 24 weeks after initial carcinogen treatment. Only 3 out of 12 trichoepitheliomas occurred singly, the rest being mixed tumours with squamous cell carcinomas or rhabdomyosarcomas.

### DISCUSSION

The spontaneous tumour incidence in the mouse strain used in this study is very low, and includes alveolar carcinomas and inflammatory changes in the ovary. MCA as well as PMA and acetone in the concentrations employed in this study, did not increase the incidence of spontaneously occurring tumour types.

The promoting range of TPA (tetradecanoyl phorbolacetate) or PMA has been found to be 0·0016–0·016 $\mu$mol applied per week or biweekly (Raick *et al.*, 1972; Baird and Boutwell, 1971). Application of phorbol esters to initiated mouse skin increases the number of papillomas induced as a function of time, the promoting action being dose-dependent (Baird and Boutwell, 1971; Raick *et al.*, 1972; Van Duuren *et al.*, 1973) and several palpable tumours develop in a single mouse. When MCA is injected s.c., a single palpable growth containing more than one histopathological tumour type is often obtained (Bhisey and Sirsat, 1966). PMA-treated mice also develop a single growth with mixed histopathology at the site of carcinogen injection. However, the earlier appearance of sebaceous cysts, rhabdomyosarcomas and squamous cell carcinomas in PMA-treated mice, when compared to those in mice injected MCA alone, indicates that this tumour promoter hastens the progression of precancerous cells into frankly malignant ones, by providing a selective proliferative stimulus.

As observed in this study, trichoepithelioma induction requires long promotion treatment, lasting up to 18 weeks. Of the 12 tumours of this type, 3 appeared between 8 and 12 PMA applications while the remainder developed after 13 to 36 applications of PMA. Trichoepitheliomas which develop as a result of enhanced differentiation of epidermal cells into hair follicles are not known to undergo malignant changes (Hashimoto and Lever, 1971) and do not occur in MCA-injected mice in the absence of promotion with PMA. This indicates that s.c. MCA provides a feeble promoting stimulus with respect to epidermal carcinogenesis. Topical application of PMA effectively transforms the initiated epidermal cells into a benign lesion.

Focal areas of extensive fibroblastic proliferation in mouse skin treated with PMA were observed by Raick *et al.* (1972). Sivak (1974) has shown that PMA causes preferential outgrowth of SV40-transformed 3T3 cells in the presence of a large number of untransformed cells. He suggested that a similar situation may occur *in vivo* in mouse skin. The reasons for the total inhibition of fibrosarcomas on PMA application are not understood. This phenomenon points to the initiation by PMA of molecular events which render the promoting influence of MCA ineffective, depriving precancerous fibroblasts of their rapid selective growth potential. An increase in the number of rhabdomyosarcomas, and their early occurrence on exposure to the tumour-promoter, show that PMA causes selective proliferation of muscle cells rendered precancerous by MCA.

The tumour induction data obtained in this study favours the multiphasic process of s.c. induced chemical carcinogenesis in the mouse skin. Although epithelial cells, fibroblasts as well as muscle cells, are transformed to a precancerous state 6 weeks after exposure to MCA, PMA application results in early selective propagation of precancerous epithelial and muscle cells into malignant tumours, and complete inhibition of fibrosarcomas. In the induction of trichoepitheliomas, however, both phases of tumour, promotion appear to be involved. The modified tumour induction pattern obtained on PMA treatment suggests that in a tissue, although different cell types may be transformed by a carcinogen, the promoting stimulus decides decisively which of these will progress further into a frank neoplasm.

### REFERENCES

Baird, W. M. & Boutwell, R. K. (1971) Tumor promoting Activity of Phorbol and Four Diesters of Phorbol in Mouse Skin. *Cancer Res.*, **31**, 1074.

Berenblum, I. (1954) Speculative Review: The Probable Nature of Promoting Action and its Significance in the Understanding of the Mechanism of Carcinogenesis. *Cancer Res.*, **14**, 471.

Berenblum, I. & Shubik, P. (1947) The Role of Croton Oil Applications Associated with a Single Painting of a Carcinogen in Tumour Induction of the Mouse Skin. *Br. J. Cancer*, **1**, 379.

Bhisey, R. A. & Sirsat, S. M. (1966) Methylcholanthrene Carcinogenesis in the Swiss Albino Mouse in Relation to Differential Oncogenesis of Skin Tumours. *Br. J. Cancer*, **20**, 418.

Hashimoto, K. & Lever, W. (1971) In *Dermatology in General Medicine*. Ed. T. B. Fitzpatrick. McGrew-Hill Book Company. p. 440.

Hecker, E. (1968) Cocarcinogenic Principles from the Seed Oil of Croton Tiglium and from other Euphorbiaceae. *Cancer Res.*, **28**, 2338.

Hennings, H. & Boutwell, R. K. (1970) Studies on the Mechanism of Skin Tumor Promotion. *Cancer Res.*, **30**, 312.

Raick, A. N., Thumm, K. & Roy Chivers, B. (1972) Early Effects of 12-O-tetradecanoyl-phorbol-13-acetate on the Incorporation of Tritiated Precursor into DNA and the Thickness of the Interfollicular Epidermis and their Relation to Tumour Promotion in Mouse Skin. *Cancer Res.*, **32**, 1562.

Raick, A. N. (1973) Ultrastructural, Histological and Biochemical Alterations Produced by 12-O-tetradecanoyl phorbol-13-acetate on Mouse Epidermis and their Relevance to Skin Tumour Promotion. *Cancer Res.*, **33**, 269.

Salaman, M. H. (1952) The Latent Period of Co carcinogenesis. *Br. J. Cancer*, **6**, 155.

Sivak, A. (1974) Action of Tumour Promoting Agents in Cell Culture Systems. *Abstracts XIth International Cancer Congress* **2**, 64.

Van Duuren, B. L. (1969) Tumor Promoting Agents in Two Stage Carcinogenesis. *Prog. exp. Tumor Res.*, **11**, 31.

Van Duuren, B. L., Sivak, A., Segal, A., Seidman, I. & Katz, C. (1973) Dose Response Studies with a Pure Promoting Agent, Phorbol Myristate Acetate. *Cancer Res.*, **33**, 2166.